# Militarization, human rights violations and community responses as determinants of health in southeastern Myanmar: results of a cluster survey

**DOI:** 10.1186/s13031-015-0059-0

**Published:** 2015-10-06

**Authors:** William W. Davis, Luke C. Mullany, Matt Schissler, Saw Albert, Chris Beyrer

**Affiliations:** Center for Public Health and Human Rights, Johns Hopkins Bloomberg School of Public Health, Baltimore, MD USA; Department of Anthropology, University of Michigan, Ann Arbor, MI USA; Karen Human Rights Group, Mae Sot, Thailand

**Keywords:** Human Rights, Burma, Myanmar, Militarization, Agency, Resilience, Protection, Self-Protection, Public Health

## Abstract

**Background:**

The Myanmar army and ethnic armed groups agreed to a preliminary ceasefire in 2012, but a heavy military presence remains in southeastern Myanmar. Qualitative data suggested this militarization can result in human rights abuses in the absence of armed engagements between the parties, and that rural ethnic civilians use a variety of self-protection strategies to avoid these abuses or reduce their negative impacts. We used data from a household survey to determine prevalence of select self-protection activities and to examine exposure to armed groups, human rights violations and self-protection activities as determinants of health in southeastern Myanmar.

**Methods and findings:**

Data collected from 463 households via a two-stage cluster survey of conflict-affected areas in eastern Myanmar in January 2012, were analyzed using logistic regression models to identify associations between exposure to state and non-state armed groups, village self-protection, human rights abuses and health outcomes. Close proximity to a military base was associated with human rights abuses (PRR 1.30, 95 % CI: 1.14-1.48), inadequate food production (PRR 1.08, 95 % CI: 1.03-1.13), inability to access health care (PRR 1.29, 95 % CI: 1.04-1.60) and diarrhea (PRR 1.15, 95 % CI: 1.05-1.27. Direct exposure to armed groups was associated with household hunger (PRR1.71, 95 % CI: 1.30-2.23). Among households that reported no human rights abuses, risk of household hunger (PRR 5.64, 95 % CI: 1.88-16.91), inadequate food production (PRR 1.95, 95 % CI: 1.11-3.41) and diarrhea (PRR 2.53, 95 % CI: 1.45-4.42) increased when neighbors’ households reported experiencing human rights abuses. Households in villages that reported negotiating with the Myanmar army had lower risk of human rights violations (PRR 0.91, 95 % CI: 0.85-0.98), household hunger (PRR 0.85, 95 % CI: 0.74-0.96), inadequate food production (PRR 0.93, 95 % CI:0.89-0.98) and diarrhea (PRR 0.89, 95 % CI:0.82-0.97). Stratified analysis suggests that self-protection strategies may modify the effect of exposure to armed groups on risk of human rights violations and some health outcomes.

**Conclusion:**

Militarization may negatively affect health in southeastern Myanmar, and village self-protection activities may reduce these impacts. As southeastern Myanmar opens to international health and development interventions, implementing agencies should consider militarization as a determinant of health and design interventions that can mediate its effects. Such interventions should take into account existing self-protection strategies, seek to provide support where possible and, at all times, take care not to unintentionally undermine them.

**Electronic supplementary material:**

The online version of this article (doi:10.1186/s13031-015-0059-0) contains supplementary material, which is available to authorized users.

## Background

Southeastern Myanmar, encompassing eastern Bago region, Mon and Karen states, and Tanintharyi Region, bordering Thailand, has for decades been made up of a patchwork of state and ethnic armed groups seeking to administer overlapping territories [1]. During 60 years of conflict in south eastern Myanmar, human rights violations have had detrimental effects on health, including direct injury, loss of food supplies, malnutrition, inability to access healthcare and loss of capital used to produce health [2–6].

In early 2012 the Government of Myanmar began agreeing to preliminary ceasefires with these groups and, though the negotiation process remains ongoing, this has resulted in a significant reduction in armed conflicts – though not a complete cessation. Reports from these areas over the last two decades, however, indicate that militarization, or the presence of the armed actors, even in the absence of armed clashes can result in human rights violations, including forced labour, capricious taxation, and land grabbing; arbitrary arrest, detention and execution; forced relocation and movement restrictions; and sexual violence [7–9]. This suggests that, even following ceasefires or the otherwise cessation of violent clashes in the region, civilians’ health and human rights may still be affected by the presence of the military [10].

Southeastern Myanmar is an example of a region in which international interventions to protect civilians from conflict and armed groups have had mixed results. This is primarily because, throughout the period of armed conflict (1948–2012), international humanitarian protection actors were denied access to civilian populations. In comparison to international efforts, it has been widely recognized that local strategies to manage risks associated with conflict and militarization can be extremely effective, though they should not be taken as a substitute for parties to conflict meeting their responsibilities to respect obligations under human rights and humanitarian law. The Active Learning Network for Accountability in Practice (ALNAP) guide for humanitarian protection, for example, notes that “Humanitarian common sense affirms the value of people's own knowledge, capacity, insight and innovation in any given situation that threatens them… People are seldom passive when they feel at risk: they engage in a range of finely judged actions to cope, respond, adapt and survive” [11]. Critics also note, however, that top-down international approaches rarely incorporate civilians’ management of their own risks or inform civilians how best to manage risk [12–14].

Local self-protection strategies have been well-documented in southeastern Myanmar. Beginning in the early 2000s, local organization the Karen Human Rights Group (KHRG) also began seeking to document individual and village-level self-protection strategies and then develop interventions to strengthen the most effective approaches [8]. Building on this, the *Local to Global Protection Project*, began additional research on these strategies and potentials for international support [9]. These studies identified multiple effective strategies used by villagers to protect themselves both from abuse associated with clashes between armed groups (e.g., targeted attacks on civilians, indiscriminate fire) and those abuses that occur in light of militarization, particularly administration of territory by the state army rather than civilian authorities (forced labour and capricious taxation; arbitrary arrest, detention and execution; forced relocation and movement restrictions). These strategies can be organized into the three-part typology, which categorizes self-protection strategies as *containing*, *avoiding*, or *confronting* protection threats [15]. In southeastern Myanmar, examples of these strategies have ranged from negotiating with armed groups, fleeing the village to avoid abuse, to using homemade landmines and *Gher der* (“home guard” militias) to protect villages [7–9]. Strategies that are assessed by this research, which represent just a few of the self-protection activities people use, are fleeing, refusing to comply, negotiating and paying bribes to reduce goods or labor demanded by armed groups.

Following the end of a major military offensive during 2005–2008, levels of armed conflict in southeast Myanmar waned substantially, though some localized areas continued to see fighting through the end of 2011 [7, 16]. Initial ceasefires in early 2012, along with political liberalizations in the Myanmar government, have led to dramatically increased international development aid for Myanmar, including in the southeast. As previous governments in Myanmar restricted access of humanitarian groups to conflict zones, experience working in these areas is largely limited to community-based groups. Challenges for new development efforts will include understanding determinants of health unique to southeastern Myanmar, where conflict has abated yet militarization persists.

We analyzed associations between exposure to armed groups and exposure to human rights violations as predictors of low food security and poor health outcomes, and we examined self-protection strategies as modifiers of these relationships.

## Methods

The data for these analyses were collected through a multi-stage cluster survey conducted in southeastern Myanmar in 2012 initially designed to identify household-level associations between human rights violations and health outcomes [17]. In this analysis, information collected at the household level (human rights violations, exposures to armed groups, health outcomes and community self-protection activities) was used to create village-level variables (any or no community self-protection activities), combined with data collected from village leaders (distance to military base) to examine determinants of health. We examined associations between militarization and human rights violations, militarization and health outcomes, human rights violations and health, and effect modification of self-protection on these associations.

### Survey instrument

The survey questionnaire comprised four modules that assessed demographics and health of household members, access to health care, food security and human rights violations. One respondent for each household answered for all individuals in the household. The first module covered age and sex distribution of household members, middle-upper arm circumference of children under five, night blindness and diarrhea (both in the two weeks prior to the survey) in all household members. The next module covered access to health care in southeast Myanmar. The third module covered food security in two sections: the six-question USAID Food and Nutrition Technical Assistance Project (FANTA) household hunger (HHH) survey, which covered one month prior to the survey, and the months of adequate household food production (MAHFP) survey [18, 19]. Months of adequate household food production is a count of the number of months in the preceding year that the household had sufficient food. The final module covered human rights violations, exposure to armed groups and self-protection activities, all with a recall period of the year before the survey. Six responses were possible for the village self-protection question, based on what community groups said were most common and what could be most accurately captured with this survey instrument: negotiate (with armed groups for reductions in demands of goods or labor), pay (to reduce labor/goods demanded), flee (to avoid complying with demands), refuse (to comply with demands), no response and don’t know.

### Sampling

The sampling universe for the original survey, shown on a previously published map [17], included 80,000 adults and children living in 250 villages in clinical catchment areas served by community-based health organizations in southeastern Myanmar. Implementing partners provided population data from the areas where they worked. No major population movements were reported in these areas during the time covered by the survey. Some forced displacement did result from conflict land confiscations, and economic migration was likely ongoing, but we do not feel that these movements would significantly change the implementing partners’ population figures [20].

According to sample size calculations for the original survey, we needed to approach 720 households in order to estimate with 5 % precision prevalence of human rights violations on the order of 15 %. The calculation assumed a survey return rate of 82 % (based on previous surveys in the research area) [4, 5, 21–23] and a design effect of 3.0 (because human rights violations typically cluster within communities at higher rates than other outcomes more commonly measured in health surveys). To reach 720 households, we undertook a 90 cluster x 8 household per cluster design; this many-clusters-of-small-size approach was followed to minimize design effect (presuming uneven distribution of outcomes across clusters), to reduce the impact on data if a surveyor lost data forms if he or she had to suddenly flee the village, to minimize the time each surveyor spent in a village (8 interviews took two days) and to balance logistical constraints of travel time between villages. In the first stage of sampling, villages were enumerated and selected randomly with probabilities proportional to each village’s population. In the second stage, eight households in each village were selected randomly using the Expanded Programme on Immunisation (EPI) method, which entails locating the approximate center of the village, randomly selecting a direction, randomly selecting one house along that direction between the center and edge of the village, and sampling that house and the seven closest houses [5, 24–30].

### Instrument development and surveyor training

The survey was based on a questionnaire used previously in Chin State, Myanmar [31] that was modified by local partners to accurately capture events in southeastern Myanmar [32]. The modification process involved extensive consultation with community-based human rights groups; this led to inclusion of questions that enabled us to analyze the relationships between militarization, abuses and self-protection strategies. The survey instrument was translated into Sgaw Karen and Burmese and then back-translated to English to ensure accuracy. The survey questionnaire covered exposure to armed groups, human rights violations, distance to the nearest Myanmar army base, and self-protection activities. Six responses were possible for the village self-protection question: negotiate reduction in labor demanded, pay to reduce the labor demanded, flee to avoid forced labor, refuse to do the labor demanded, no response and don’t know.

Partner community-based organizations contributed 22 surveyors (16 male, six female, 20–38 years old) who were working inside the study area, fluent in Burmese or Sgaw Karen and had extensive political and geographical knowledge of the region in which they worked. For security reasons, they were assigned clusters only in their home regions. Surveyors were trained in lectures and practice sessions on taking informed consent, the survey instrument and measuring middle-upper arm circumference (MUAC) of children under five over two weeks. They were required to pass an exam before implementing the survey.

### Implementation

Surveyors conducted the study in January 2012, and the questionnaire covered events that occurred 12 months prior to the survey, with the exception of the household hunger section (one month recall) and health status questions (two weeks recall). Surveyors first obtained consent from village leaders and asked about exposure to armed groups and health problems, then interviewed households. Participants were 18 years old or older (15 if married) living in the survey area, who spoke Burmese or Sgaw Karen, gave informed consent and were deemed by the surveyors to be psychologically competent to participate in the survey. Anyone who ate meals in the house for two months preceding the survey was included as a household member.

### Statistical analysis

Only households in areas affected by conflict and militarization (everywhere but Dawei/Tavoy area in the original survey, which we assumed was non-conflict and administered by civilian authorities) were included in this secondary analysis; 463 households of the original households sampled (66.5 %) were located in conflict-affected areas and only these were included in the analysis for this paper. While the original survey was sufficient to provide approximately 5 % precision around human rights violations occurring at 15 % frequency, the reduced sample size available for this secondary analysis reduces that precision to approximately 6.2 %. Data (outcomes and exposures) were weighted at either the clinical area level or the village level by population, and all analyses were performed with STATA 13 using svy commands to apply Taylor linearization, which enables conservative estimation of variance when clusters are of different sizes (as in this case). Survey coverage and participation rates were estimated. Prevalence of human rights violations, health and nutrition outcomes, and self-protection activities were estimated and 95 % confidence intervals were calculated.

**Fig. 1:**
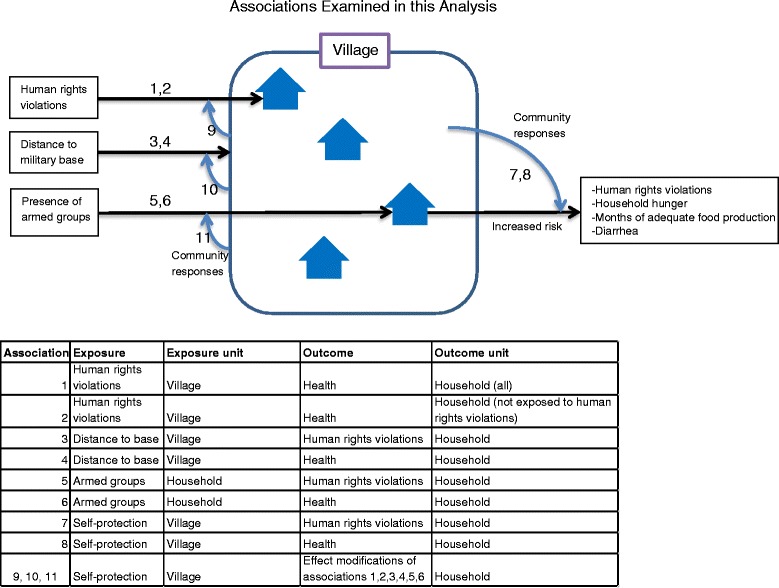
Associations Examined in this Analysis

The goal of the interpretive analysis was to quantify associations (numbered 1 through 10 in Fig. 1) of determinants of health and to examine self-protection as a modifier of the effects of those determinants. All interpretive analyses were adjusted for household size, area, type of water supply, religion, ethnicity, female-headed household and terrain (mountain or plains) using stepwise forward selection with p < 0.10 as selection criteria; unadjusted models were also done. Prevalence rate ratios between exposure to armed groups, human rights violations and health outcomes and 95 % confidence intervals were estimated using generalized linear models with log link functions or Poisson regression when models did not converge. We coded household hunger (moderate/severe and none/mild), months of adequate household food production (1–9 months/ 10–12 months) and diarrhea and night blindness (present/ not present) and human rights violations (any/none) as binary variables. The months of adequate household food production cutoff of nine months best captured the difference between households that experienced human rights violations and those that did not. About 15 % of the questions on self-protection and distance to base were missing, likely because of the sensitive nature of the questions. We used listwise deletion to handle missing data.

We first investigated militarization as a risk factor for human rights violations, using distance to Myanmar military base (defined in terms of time spent walking between the location of residence and military bases; time walking was used because foot travel was the primary mode of travel and mountainous topography means linear distances are rarely referenced by populations living in the research area) or exposures (defined as seeing any armed group, seeing specific armed groups, and the total number of different armed groups seen) as predictors (associations 3 and 5, Fig. 1).

Next we examined determinants of household health. We estimated the risks of diarrhea, household hunger (HHH), being sick and not able to access treatment (sick and no tx), and nine or fewer months of adequate household food production (MAHFP) using proximity to the nearest military base or exposure to armed groups as predictors (associations 4 and 6, Fig. 1). In a subpopulation of households that did not report human rights violations, we estimated the risk of poor health if other households in the same village reported human rights violations (associations 1 and 2, Fig. 1).

Finally we investigated the effects of self-protection on human rights violations and on health. For each village sampled, we combined the responses for household self-protection and, for the whole village, generated continuous village self-protection variables. For each village the total number of any single self-protection activity (e.g. negotiation) was between 0 and 8, the maximum number of households surveyed in the village. According to existing research reverenced above, interactions between armed groups and civilians tend to be done at a village level, for example, armed groups will demand a certain number of porters of bags of rice from a village, and the village must decide how this burden will be distributed among households. Armed groups less frequently make specific demands of a single household. Because civilians tends to interact with armed groups as a village unit, treating self-protection as a village-level continuous variable most accurately reflected the dynamics between armed groups and civilians in southeastern Myanmar.

We examined associations between village self-protection and exposure to armed groups, human rights violations and health outcomes all measured at the household level (associations 7, 8, 9, 10, 11 in Fig. 1). Next we stratified the dataset by village self-protection and examined associations between exposure to armed groups and outcomes of human rights violations or health. Small sample sizes precluded an analysis of interaction terms in multiple logistic regression.

This study was approved by the Institutional Review Board at Johns Hopkins Bloomberg School of Public Health, the Ethics Review Board at Physicians for Human Rights, and an ad hoc Karen community advisory team.

## Results and discussion

**Fig. 2:**
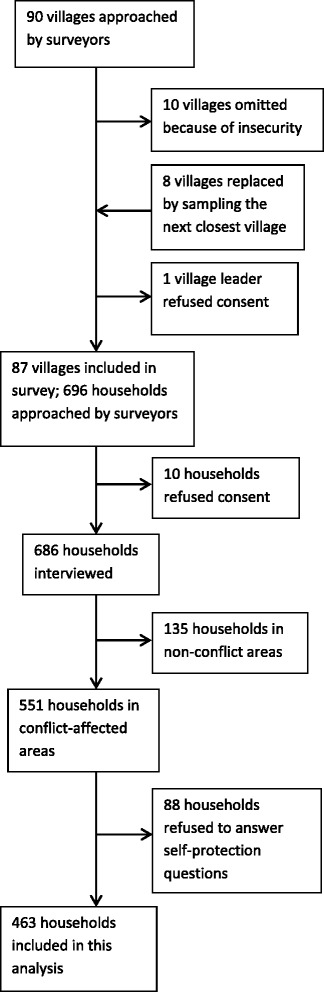
Sampling Scheme

Sampling is shown in Fig. 2. Surveyors approached 90 villages, omitted 10 because of security reasons, and substituted eight by selecting the villages closest to the insecure villages. One village leader refused to consent to the survey, and 88 villages were included in the original analysis. Of the households approached, 9 did not consent to the survey. For the sub-analysis described here, we included 551 households that were located in militarized and conflict-affected areas. We dropped 88 (16 %) households from the analysis because they refused to answer the self-protection questions, thus 463 households were included in the final analysis. This sample included 2471 people.

Health, human rights and self-protection outcomes and exposures to militarization are shown in Table 1. Of 463 households surveyed, 148 (26.8 %, 95 % CI: 20.4-34.2) reported experiencing at least one human rights violation, 354 (79.7 %, 95 % CI: 72.7-85.3) reported seeing at least one armed group, and 226 (48.7 %, 95 % CI: 38.4-59.3) reported engaging in at least one self-protection activity in the year prior to the survey. The distance between surveyed household and the nearest army base ranged from 1 to 30 h hiking.

**Table 1:** Summary of human rights abuses, self-protection activities and militarization measures

Indicator	Cases	Total	Percent	95 % CI
Human Rights Violations				
Any household member reporting human rights violations	148	463	26.8	20.4–34.2
Any household member reporting forced labor	129	463	22.1	16.8–28.7
Health				
Moderate/severe household hunger	57	459	13.5	8.7–20.2
<10 months of adequate household food production	185	460	38.4	30.0–47.5
Any household member reported sick and unable to access treatment	58	458	11.7	7.4–18.1
Any member of the household reporting diarrhea	167	2424	7.9	5.5–11.1
Self-protection				
Negotiate	138	463	33.2	24.2–43.6
Pay	129	463	22.8	17.1–29.6
Leave	12	463	3.4	1.2–9.2
Refuse	68	463	19.8	12.4–30.1
Any	226	463	48.7	38.4–59.3
Armed groups seen by households				
None	109	463	20.3	14.7–27.3
At least one	354	463	79.7	72.7–85.3
Myanmar Army	148	463	31.2	23.7–41.5
Myanmar Army or BGF	179	463	35.7	27.2–45.2
Total number of different armed groups seen by household				
0	109	457	20.4	14.8–27.5
1	194	457	48.8	39.1–58.6
2	91	457	21.6	14.1–31.7
3	51	457	7.6	4.6–12.2
4	12	457	1.5	0.6–4.0
Distance to Army base, in hours hiking				
1	120	403	25.9	16.9–37.7
2	60	403	19.2	10.7–32.1
3	98	403	21.1	15.3–35.8
4	39	403	7.3	3.2–12.7
5	32	403	6.4	2.6–14.9
6	8	403	1	0.2–4.9
10	14	403	4.7	1.3–15.5
12	16	403	5.3	1.5–16.4
15	9	403	3.4	0.1–14.8
30	7	403	2.7	0.1–13.7

We identified several statistically significant associations between militarization, human rights violations and poor health outcomes (Table 2). Proximity to military bases was associated with increased risk of human rights violations and poor health outcomes. For each hour of hiking closer to a Myanmar army base (relative to responding household’s location), a household’s risk of experiencing any human rights violation increased by 30 % (1.30, 95 % CI: 1.14-1.48). Similarly, risk of reporting <10 months of adequate household food production increased by 8 % (1.08, 95 % CI: 1.03-1.13), risk of a household member being sick and not able to receive treatment increased by 29 % (01.29, 95 % CI: 1.04-1.60), and risk of a household member reporting diarrhea increased by 15 % (1.15, 95 % CI: 1.05-1.27). For each of the outcomes, design effects were calculated; these ranged from 0.8 to 2.1.

**Table 2:** Associations between militarization, human rights violations and health

Exposures to militarization	Health and human rights outcomes									
	Any household member reporting human rights violations			No household members reporting human rights violations			Unadjusted model		Adjusted model	
	exposed^1^	cases^2^	%	exposed^3^	cases^4^	%	PRR	95 % CI	PRR	95 % CI
Distance to base							1.3	1.14–1.48	-	-
Saw any armed group	354	133	37.6	109	15	13.8	3.39	1.53–7.55	1.13^a^	0.58–2.19
Saw MM Army	148	60	40.5	309	85	27.5	1.42	0.85–2.40	1.59^a^	0.92–2.75
Saw MM Army or BGF	179	69	38.5	278	76	27.3	1.41	0.84–2.39	1.6^a^	0.93–2.77
	Any household member reporting forced labor			No household members reporting forced labor			Unadjusted model		Adjusted model	
	exposed	cases	%	exposed	cases	%	PRR	95 % CI	PRR	95 % CI
Distance to base							1.28	1.13–1.46	-	-
Saw any armed group	354	116	32.8	109	13	11.9	3.18	1.30–7.83	-	-
Saw MM Army	148	52	35.1	309	75	24.3	1.35	0.79–2.30	1.53^a^	0.86–2.72
Saw MM Army or BGF	127	61	48	330	118	35.8	1.39	0.81–2.38	1.57^a^	0.88–2.79
	Moderate/Severe Household Hunger			Low Household Hunger			Unadjusted model		Adjusted model	
	exposed	cases	%	exposed	cases	%	PRR	95 % CI	PRR	95 % CI
Distance to base							1.22	0.99–1.49	-	-
Saw any armed group	351	50	14.2	108	7	6.5	1.86	0.74–4.73	2.06^b^	0.88–4.82
Saw MM Army	148	40	27	305	16	5.2	5.59	2.51–12.44	6.01^b^	2.73–13.25
Saw MM Army or BGF	178	42	23.6	275	14	5.1	5.2	2.28–11.89	5.69^b^	2.48–13.02
	<10 months of adequate household food production			10–12 months of adequate household food production			Unadjusted model		Adjusted model	
	exposed	cases	%	exposed	cases	%	PRR	95 % CI	PRR	95 % CI
Distance to base							1.08	1.03–1.13	-	-
Saw any armed group	352	149	42.3	108	36	33.3	1.1	0.77–1.59	1.32^b^	0.72–2.41
Saw MM Army	147	51	34.7	307	132	43	0.72	0.48–1.07	-	-
Saw MM Army or BGF	178	60	33.7	276	123	44.6	0.7	0.46–1.05	0.78^b^	0.52–1.18
	Any household member reported sick and unable to access treatment			No household member reported sick and unable to access treatment			Unadjusted model		Adjusted model	
	exposed	cases	%	exposed	cases	%	PRR	95 % CI	PRR	95 % CI
Distance to base							1.29	1.04–1.60	-	-
Saw any armed group	349	53	15.2	109	5	4.6	3.3	1.09–9.91	3.57^b^	1.27–10.02
Saw MM Army	147	44	29.9	305	13	4.3	8.89	3.91–20.15	11.4^b^	5.18–24.88
Saw MM Army or BGF	176	48	27.3	276	9	3.3	11.01	4.33–28.34	12.46^b^	5.06–30.69
	Any member of the household reporting diarrhea			No member of the household reporting diarrhea			Unadjusted model		Adjusted model	
	exposed	cases	%	exposed	cases	%	PRR	95 % CI	PRR	95 % CI
Distance to base							1.15	1.05–1.27	-	-
Saw any armed group	1847	140	7.6	577	27	4.7	1.51	0.80–2.84	-	-
Saw MM Army	800	64	8	1596	102	6.4	1.13	0.58–2.19	-	-
Saw MM Army or BGF	950	69	7.3	1446	97	6.7	1.02	0.54–1.90	1.12^b^	0.60–2.10

^a^adjusted for mountainous terrain

^b^adjusted for mountainous terrain, source of drinking water

^1^Number of households exposed to “militarization” e.g. saw any armed group

^2^Number of households that were exposed to militarization that also reported a human rights violation

^3^Number of households that were not exposed to any armed group

^4^Number of households not exposed to any armed group but that did report a human rights violation

Exposure to armed groups was also associated with increased risk of a household experiencing human rights violations and poor health: households that reported seeing Myanmar army or Border Guard Forces were 5.7 times more likely to report moderate or severe household hunger (95 % CI: 2.5-13.0) and 12.5 times more likely to report that a household member was sick and not able to get treatment (95 % CI: 5.06-30.69) compared with households that did not report seeing these groups (Table 2).

Human rights violations of one household were associated with increased risk of poor health in neighboring households. Among households that did not report human rights violations, having a neighbor that reported human rights violations increased the risk of household hunger and diarrhea by 5.64 (95 % CI: 1.88-16.91) and 2.53 (95 % CI: 1.45-4.42) times, respectively, compared with households not having neighbors who reported rights violations (Additional file 1).

Of the types of self-protection assessed, village negotiation had the most statistically significant results out of all four forms assessed, and is the only type discussed here (Table 3, Additional files 1, 2, 3 and 4). Negotiation was associated with reduced risks of household hunger (PRR 0.85, 95 % CI: 0.74- 0.96), reporting < 10 months of adequate household food production (PRR 0.93, 95 % CI: 0.89-0.98), diarrhea (RR 0.89, 95 % CI: 0.82-0.97), having a household member sick and not able to access treatment (PRR 0.81, 95 % CI:0.71-0.93) and reduced risk of any human rights violation (PRR 0.91, 95 % CI: 0.85-0.98).

**Table 3:** Associations between village negotiation and human rights violations and health indicators

	PRR	95 % CI
Moderate/Severe Household Hunger	0.85	0.74 – 0.96
<10 months of adequate household food production	0.93	0.89 – 0.98
Any member of the household reporting diarrhea	0.89	0.82 – 0.97
Any household member reported sick and unable to access treatment	0.81	0.71 – 0.93
Any household member reporting human rights violations	0.91	0.85 – 0.98
Any household member reporting forced labor	0.92	0.86 – 1.00

All results were obtained using unadjusted models

Furthermore, village negotiation appeared to modify the relationship between exposure to armed groups and human rights violations and health outcomes (Additional file 3). Village-level negotiation slightly weakened the associations between exposure to armed groups and experiencing any human rights violation (no negotiation PRR 1.74 95 % CI: 1.21-2.51, negotiation PRR 1.66, 95 % CI: 1.25-2.23), suggesting that risk of experiencing human rights violations might be less in villages that negotiated. The modifying effect was stronger on the relationship between exposure to armed groups and health; the greatest differences were in exposure to the number of armed groups and household hunger (no negotiation PRR 2.53 95 % CI: 1.59-4.04, negotiation PRR 1.25 95 % CI: 0.36-4.38) and exposure to any armed group and diarrhea (no negotiation PRR 5.49 95 % CI: 1.94-15.54, negotiation PRR 0.70 95 % CI: 0.3-1.66), suggesting that, given the same exposures to armed groups, households in villages that negotiated tended to have better health outcomes than households in villages that did not negotiate. Village negotiation also modified the effect between exposure to Myanmar Army or Border Guard Forces and health, although for this exposure the effect was opposite for nine or fewer months of adequate household food production (no negotiation PRR 0.56 95 % CI: 0.35-0.90, negotiation PRR 1.26 95 % CI: 0.73-2.18) and diarrhea (no negotiation PRR 0.92 95 % CI: 0.41-2.08, negotiation PRR 1.47 95 % CI: 0.78-2.75), suggesting households in villages that negotiated with Myanmar Army or Border Guard Forces had increased risk of these poor health outcomes compared with households in villages that did not negotiate, given the same exposures to these armed groups.

### Discussion

The data presented here extends previous research on health and human rights in eastern Myanmar. It suggests that militarization could be a determinant of health, that human rights violations experienced by one household could affect health in other households in the same village, and that actions taken by households might mitigate these effects.

Health is an effective measure for examining the results of militarization, violence and human rights violations, [33–35] and conflict is a well-documented determinant of health. It is associated with increased mortality from direct violence, but it also affects distal determinants of morbidity and mortality by limiting access to healthcare, disrupting health services, depressing the economy and creating food insecurity [36–43]. The mechanisms through which militarization in southeast Myanmar could affect health might be similar to the effects of conflict on health. That hiking time to an army base is a predictor of increased risk of human rights violations suggests that abuses by the army continue to be widespread and systematic in Karen state, despite reduced conflict.

This study suggests that human rights violations experienced by one household might affect the health of a nearby household. Several mechanisms could explain this relationship: through stifling village economic production and trade (for example, when forced labor limits household productivity), by limiting movement and thus transport of medicine, by frightening witnesses of human rights violations into hiding and limiting household productivity, or by creating burdens on households that experienced rights violations that are shared by other households through social, economic and family networks. Human rights violations in neighbors’ households may also indicate a high level exposure to militarization, and the mechanisms through which militarization may affect health, such as through stifling the economy, limiting access to fields or hospitals, or suppressing the health system, may also be present.

This study confirms for the first time that self-protection strategies are widely used in this setting, which supports previously qualitative arguments that people are not ‘passive victims’ of conflict [11]. That civilians in southeastern Myanmar have the capacity to successfully use self-protection suggests that international actors in the region and in other contexts should continue to explore ways to support these activities. At a minimum, international actors should take care not to unintentionally undermine or constrain the use of such strategies, as this would then have unintended negative health outcomes.

We identified several cases in which negotiation weakened the effect of militarization on poor health outcomes, and two cases in which village negotiation strengthened the association. The discrepancy might be explained by unmeasured variables, such as proximity to roads, access to aid from CBOs, other unmeasured forms of self-protection or multiple exposures to the same armed group. The effects of self-protection on health may also involve mechanisms other than influencing the actions of armed groups. Villages that engage in self-protection may be more likely to engage in health-seeking behaviors (through self-efficacy or dignity mechanisms) or have improved mental health, both of which could affect physical health. Because villagers know the context of the conflict and (usually) the personalities of local commanders and units, they may only try to negotiate with specific armed groups with which they think there is some chance of success. Finally, self-protection may function at low-dose exposures to armed groups but, at higher doses or where the needs of armed actors are overwhelming and space for negotiation ceases to exist, they may be less effective or entail negative secondary effects [7].

This research contributes quantitative data to a growing body of evidence of successful ways in which civilians reduce risk of harm in times of conflict. Previous qualitative research in southeastern Myanmar suggests that villagers manage threats by complying with demands or negotiating with armed groups, avoiding threats by fleeing and confronting threats through public advocacy and active resistance [9]. Previous research found that paying bribes to armed groups was common and usually successful, as was selecting older women village as leaders who were seen as being better able to negotiate with armed groups, and could sometimes get compensation from the army for attacks and also prevent forced relocations [9]. Villages also said that they would under-report their labor force or amount of food supplies in order to reduce the amount of food or labor demanded [9].

Other forms of negotiation, such as political activism and bearing witness, have been documented in both conflict and militarized non-conflict settings. For example, other forms of negotiation, such as political activism and bearing witness, were documented in women’s rights groups in Afghanistan and community-based groups in Guatemala [44, 45]. In the Afghan case, community groups organized humanitarian and political activities to encourage people to resist oppression. Research found that their activities raised public awareness of oppression and resulted in stronger communities [44]. Guatemalan community groups organized health tribunals to advocate for remediation from mining companies [45].

Civilians in South Kordofan, Sudan, reported that communication with hostile forces could help to reduce the risk of attack [46]. They suggested it might increase empathy from soldiers, help soldiers to think about the consequences of their actions, or help to change the political landscape to one of peace.

Intrastate conflicts are increasingly common, and they pose new threats as civilians become displaced and access by international groups is limited or nonexistent [47]. In these situations community roles for preventing violence and reducing risks from conflict become more important [38]. As knowledge of community self-protection mechanisms increases, international actors will be better able to incorporate these mechanisms in their protection interventions [12, 46, 48]. Knowledge of specific strategies that are effective may inform the development of more successful humanitarian interventions. On the other hand, seeking to understand such strategies is important so that international actors do not unintentionally undermine civilians’ efforts. Finally, even where specific strategies have not yet been identified, recognizing the existing capacities of communities seeking to protect themselves may be the most important starting point for international actors.

This research is subject to several limitations. Limitations of cluster sampling apply to this survey and have been documented in detail [26, 27, 29, 49]. The results of this survey cannot be generalized to areas outside of the clinical catchment areas in Karen State where the implementing CBOs operate, as these areas were not included in the sampling frame.

Insecurity precluded assessment of ten of the pre-selected clusters. Systematic differences, if present, in exposures and outcomes in these villages compared with sampled villages, would likely result in an underestimation of the associations between human rights violations and health outcomes.

Sample size limited the analysis: the original survey was not powered for this analysis, and missing data was high for questions relating to self-protection activities and distance to the nearest military base. In only one village did all eight households refuse to answer any of the self-protection questions. The remainder were in 22 villages, which were concentrated in two townships (Hpa An and Hpapun). These townships had the highest proportion of households reporting seeing Myanmar army or BGF.

To further explore this, we used logistic regression to identify predictors of missing self-protection variables and found that households that had seen the Myanmar army or allied BGF (but not any armed group) were statistically less likely to respond to the question on self-protection. Missing data was not associated with health outcomes (data not shown). It is possible that people in these areas were more reluctant to answer these questions because they perceived a greater threat of reprisal from armed groups or because they wanted to keep their protection activities secret.

Qualitative data suggest that, self-protection strategies are less effective or may result in their own unintended negative consequences. If this is true, then deleting the missing data would bias the analysis towards strengthening the association between self-protection and fewer human rights violations and better health outcomes. If self-protection mechanisms continue work at high doses of exposure to armed groups, deleting the missing data would not change the parameter estimate.

The recall period for survey questions ranged from two weeks to one year, and these data are subject to recall bias. MAHFP, when broken down by month, reflected previously published data on seasonal food security trends, suggesting bias here was minimal [32, 50]. Herlihy et. al. suggest recall bias is minimal for traumatic events [51].

Respondents who experienced human rights violations may have been more likely to recall self-protection activities if a traumatic experience helped to trigger the memory. If this was the case, the analysis of self-protection and rights violations would have been biased towards showing stronger associations between human rights violations and self-protection.

Social desirability bias may be present if surveyors or participants felt they would benefit from exaggerating results that would make stronger advocacy. This issue was covered extensively during training and was also written into the informed consent in order to minimize this bias.

Although surveyors lived and worked in the areas they were assigned to cover, it is possible that interviewees were not comfortable discussing sensitive issues .such as health or human rights violations. Due to logistical constraints, we did not match surveyors and respondents by sex. During the informed consent process, surveyors assured respondents of anonymity and confidentiality, but it is possible that sensitive information was underreported.

We did not measure mental health or self-reported health, proximity to roads or rivers, or multiple exposures to the same armed group. These measurements may have enabled a more accurate analysis of determinants of health.

## Conclusions

The ongoing peace process in southeastern Myanmar has prompted a reduction in conflict and the best opportunity for genuine peace in six decades, and this is being accompanied by an influx of development and health interventions. The effects of militarization on health cannot be overlooked when planning or evaluating public health interventions in this area. Donors aiming to improve health in Myanmar must address the problem of militarization, and evaluations of public health interventions in southeastern Myanmar should include assessments of this and also of human rights violations. Rural ethnic villages have learned to cope with militarization, and international agencies should respect and support these community strategies when planning new programs.

## Additional files

Additional file 1:
**Associations between health in households that did not experience human rights violations and human rights violations in neighbors’ households.** (XLSX 10 kb) Additional file 2:
**Associations between village self-protection activities and risks of human rights violations and poor health.** (XLSX 11 kb) Additional file 3:
**Negotiation as a modifier of associations between exposure to armed groups and risk of poor health.** (XLSX 12 kb) Additional file 4:
**Negotiation as a modifier of associations between exposure to armed groups and risk of any household member reporting human rights violations.** (XLSX 10 kb)

## Abbreviations

ALNAP: Active Learning Network for Accountability in Practice in Humanitarian Action
BGF: Border Guard Force
FANTA: Food and Nutrition Technical Assistance
HHH: Household Hunger
KHRG: Karen Human Rights Group
MAHFP: Months of Adequate Household Food Production
